# Two new species of *Tribonium* Saussure, 1862 (Blattodea, Blaberidae, Zetoborinae), with a key to males of the genus

**DOI:** 10.3897/zookeys.502.8591

**Published:** 2015-05-05

**Authors:** Leonardo de Oliveira Cardoso da Silva, Sonia Maria Lopes

**Affiliations:** 1 Dept. de Entomologia, Museu Nacional, Universidade Federal do Rio de Janeiro, Rio de Janeiro, Brazil

**Keywords:** Blattodea, genitalia, key, new species, taxonomy

## Abstract

This contribution describes and illustrates the male genitalia of two new species of Blaberidae collected in the state of Minas Gerais, Brazil: *Tribonium caldensis* **sp. n.** resembles *Tribonium neospectrum* Lopes, 1978, differing in the coloration, size and genitalia; and *Tribonium morroferrensis* **sp. n.**, which resembles *Tribonium guttulosum* (Walker, 1868) but also differs in the size and coloration of specimen and genital pieces morphology. The genital plates were removed after dissection of the posterior part of the abdomen, and were stored in microvials containing glycerin, attached to the respective exemplar in the collection of the Museu Nacional of Rio de Janeiro, Brazil. A key to males of the species of the genus is also presented. Illustrations of *Tribonium neospectrum* and *Tribonium guttulosum* are provided to clarify the comparisons with the new species described here.

## Introduction

The genus *Tribonium* was described by Saussure (1862), who designated *Blatta spectrum* Eschscholtz, 1822 as the type species. Originally, Saussure described the genus as encompassing species with the following characteristics: antennae slightly longer than body; pronotum transverse; supranal plate of male enlarged; cerci short; coloration brown with scattered black spots, mainly on tegmina. Species identification within the genus is difficult, requiring morphological studies of the genitalia. Saussure described *Tribonium* in a key, where he separated the genus from *Proscratea* Burmeister, 1838, which is characterized by a shorter antenna, not reaching beyond apex of abdomen; pronotum with apical margin sinuous; tegmina broad; and sides of abdomen and extremities elevated. Saussure (1864) redescribed the genus and provided additional diagnostic characters: dilated in general shape; abdomen significantly widened, dorsoventrally flattened, with serriform ridges; subgenital plate with short styles.

Brunner von Wattenwyl (1865) described the genus *Philobora*, which was later synonymized with *Tribonium*, and characterized it by the head facing down, with vertex exposed; pronotum shaped as lid over the head, with dorsal margin forming an obtuse angle; tegmina wide basally; scapular area with folded comb; anal field triangular; wings rounded, with median vein complete; legs sturdy; abdomen flattened.

Hebard (1933) described a new species of *Tribonium* from Colombia: *Tribonium colombicum*.

Princis (1964) cataloged five species, recording the genus from Brazil, Argentina, Paraguay, Bolivia and Colombia.

Roth (1970) quoted Rehn (1932) as stating that “the genera *Zetobora*, *Lanxoblatta*, *Zetoborella*, and *Schizopilia* ... are clearly derivatives of a single phylum,” whereas *Schistopeltis* and *Tribonium* “.... typifies a distinct and clearly marked phylogenetic series.” In general, the structure of the male genitalia tends to support Rehn’s hypothesis. However, Roth separated *Zetobora* from the other three genera of his grouping because of the “relatively poorly developed L2d”, and described the genitalia of some species of *Tribonium*, which he assigned to the subfamily Zetoborinae. He characterized the genitalia of the genus as having the anterior portion of the median sclerite well developed, extended dorsally, with posterior portion extended upward; and subapical incision of right phallomere well defined at middle of hook.

In a footnote, Roth (1974) made corrections to the figures of *Tribonium conspersum* (Guérin-Méneville & Percheron, 1835) and *Tribonium spectrum* (Eschscholtz, 1822) that he had published (Roth 1970).

Roth (1974) suggested that *Tribonium* is not closely related to *Schistopeltis* Rehn, 1916, and separated the former based on the uniformly rounded shape of the pronotum. In *Schistopeltis* the pronotal margin is tapered and cropped dorsally.

Lopes (1978) revised *Tribonium*, and diagnosed the genus with the following characters: triangular head with interocular space less than distance between antennae, ocelli present; maxillary palps well developed; tegmina with marginal field extended; veins numerous and irregular; wings with marginal field narrow, subcostal vein reaching basal third of scapular field; cubital vein with numerous complete and few incomplete veins, apical triangle absent; fore femur with row of tiny spines on anteroventral margin; pulvilli well developed; claws symmetrical and without specializations; arolia moderately developed; supra-anal plate cordiform, symmetrical, subgenital plate asymmetrical, with digitiform styles.

Lopes (1978) mentioned that the genus is essentially Neotropical, with its species having an overall brown coloration with scattered black spots, mainly on the tegmina; and that the general similarities make species identification difficult, and further morphological studies of the genitalia are needed. Lopes (1978) added that members of *Tribonium* are distributed from French Guiana, Colombia, Brazil, Bolivia, and Paraguay to Argentina.

Grandcolas (1993) included *Tribonium* in his phylogenetic study of the subfamily Zetoborinae, and described the species *Tribonium guyanense* based on a female. Roth (2003) listed the species that had been described after the publication by Princis (1964), and supported the idea that the genus belongs in Zetoborinae.

Lopes and Silva (2010) described a new species from the state of Amazonas, Brazil, contributing to the understanding of the genus and its geographical distribution.

Valverde et al. (2012), in their morphological study of individuals belonging to *Schistopeltis lizeri* Rehn, 1928, commented on their similarity with species of *Tribonium*, from which they differ in the configurations of the median sclerite and the pronotum.

Eight of the 11 presently known species are from Brazil: *Tribonium conspersum* (Guérin & Percheron), *Tribonium conspurcatum* (Burmeister), *Tribonium delicatum* Lopes, *Tribonium elegans* (Brunner), *Tribonium guttulosum* (Walker), *Tribonium litoris* Lopes, *Tribonium neospectrum* Lopes, and *Tribonium spectrum* (Eschscholtz).

In this contribution two new species of *Tribonium* are added, from the state of Minas Gerais, Brazil, and present illustrations of their internal genitalia and a key for the available males of the genus. *Tribonium guyanense*, *Tribonium conspurcatum*, and *Tribonium elegans* are not included in the key because they were described based only on females.

## Materials and methods

The type species (*Tribonium spectrum*) was not used in the key because the description by Saussure provides few morphological characters. Unfortunately, we received no response to our request for the loan of the type material, because of the absence of a researcher in the Moscow Museum where it is deposited. *Tribonium colombicum* was not examined because the Philadelphia Academy of Natural Sciences failed to respond to our request for the loan of the specimen deposited under number 1215. It is necessary to include color characters in the key, because these are essential for the identification of species of *Tribonium*, as is the shape of the genitalia.

The genital plates were removed after dissection of the posterior part of the abdomen, using traditional dissection techniques as described by Lopes and Oliveira (2000). After analysis, the genital plates and pieces were stored in microvials containing glycerin, and attached to the respective exemplar, a procedure developed by Gurney et al. (1964). The terminology for the genitalia and the taxonomic classification follow Roth (2003). The specimens were also compared with other specimens of *Tribonium* deposited in the Blattodea Collection of the Museu Nacional of Rio de Janeiro (MNRJ), and with descriptions in the literature.

After observation and comparison of specimens of different species of *Tribonium* under the stereomicroscope, distinguishing characteristics were selected for use in the key.

The holotypes are deposited in the collection of the Department of Entomology at the Museu Nacional of Rio de Janeiro (MNRJ).

## Results

### Tribonium caldensis sp. n.

Taxon classificationAnimaliaBlattodeaBlaberidae

http://zoobank.org/037F4C0B-C7EC-4953-A639-C7EAECFB9449

Figs 1–8

#### Type-material.

Holotype ♂, BRAZIL, Minas Gerais, Poços de Caldas, Morro do Ferro, 07/IX/1967, J. Becker, O. Roppa, O. Leoncini cols.

#### Etymology.

The species name refers to the city of Poços de Caldas, where the material was collected.

#### Description.

Dimensions (mm): Male holotype, total length 23.2; length of pronotum 4.2; width of pronotum 7.9; length of tegmen 19.8; width of tegmen 6.5.

**Figures 1–8.**
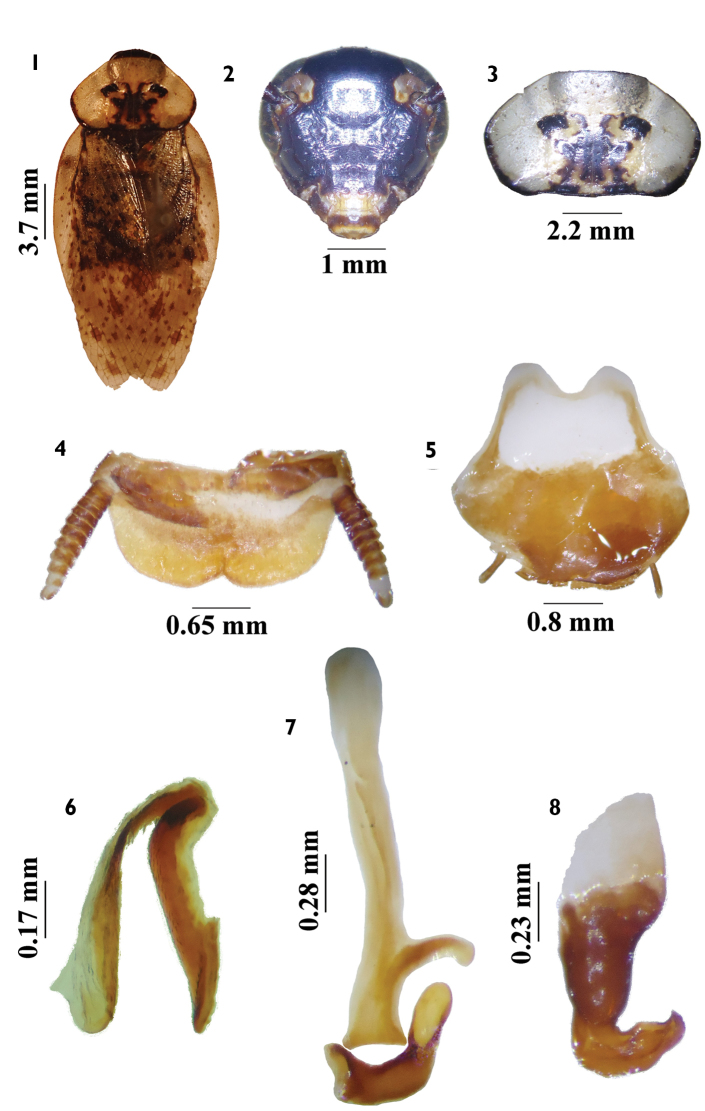
*Tribonium caldensis* sp. n. male **1** *habitus*, dorsal view **2** head, ventral view **3** pronotum, dorsal view **4** supranal plate, dorsal view **5** subgenital plate, ventral view **6** left phallomere, dorsal view **7** median sclerite, dorsal view **8** right phallomere, dorsal view.

**Male holotype.** Coloration yellowish brown with dark brown spots (Fig. 1). Head black with yellowish brown ocellar and clypeal areas (Fig. 2). Pronotum medially with symmetrical dark brown spots of no definite shape (Fig. 3). Apex of anal field of tegmina pale with brown spots. Marginal fields and scapular with brown spots. Pulvilli light brown. Arolia and claws dark brown.

**Head.** Triangular; vertex slightly exposed; interocular space ample, 1.6 mm, wider than 2/3 distance between antennae. Antenna not reaching apex of tegmen. Palps and antennae tomentose; second segment of maxillary palps small, third segment 0.71 mm in length, fourth segment same size as second, fifth segment clavate, similar in size to third segment.

**Thorax.** Pronotum convex, transverse, apical surface embossed, with rounded edges and straight basal surface. Tegmina developed, extending beyond apex of cerci; marginal field short and slightly concave; scapular field tapering toward apex, mid-field discoidal, extended apically, slightly angular along veins. Wings with marginal field narrow, subcostal vein reaching basal third of scapular field; cubital vein with numerous complete and few incomplete veins; apical triangle absent. Legs short, robust, inner side of femora with narrow posterior apical projection. Fore, mid and hind femora sparsely ciliate along anteroventral margin. Pulvilli small, present on fourth tarsal segments; claws simple, symmetrical, with concave arolia.

**Abdomen.** Supranal plate ciliated, with prominent median edges between enclosures and with slight invagination in middle portion; cerci short, ciliated, with 11 segments (Fig. 4). Subgenital plate asymmetrical (Fig. 5). Right phallomere hook-shaped, with subapical slit (Fig. 6); median sclerite with pre-apical lateral projection and apical sclerite elongated (Fig. 7); left phallomere slightly sclerotized (Fig. 8).

#### Diagnosis.

*Tribonium caldensis* sp. n. resembles *Tribonium neospectrum* Lopes, 1978 in the coloration of the head (Figs 2, 26), pronotum (Figs 3, 27), differing in the coloration of the tegmina (Figs 1, 25) and the configuration of the genital parts (Figs 6–8, 30–32). The supranal and subgenital plates are the blaberoid type (Figs 4–5, 28–29).

### Tribonium morroferrensis sp. n.

Taxon classificationAnimaliaBlattodeaBlaberidae

http://zoobank.org/D39A1E8A-8BD5-4244-81A4-64C4862E49F9

Figs 9–16

#### Type material.

Holotype ♂, BRAZIL, Minas Gerais, Poços de Caldas, Morro do Ferro, 07/IX/1967, J. Becker, O. Roppa, O. Leoncini cols.

#### Etymology.

The species name refers to Morro do Ferro, where the material was collected.

#### Description.

Dimensions (mm): Male holotype, total length 25.3; length of pronotum 4.6; width of pronotum 8.2; length of tegmina 21.6; width of tegmina 7.7.

**Figures 9–16.**
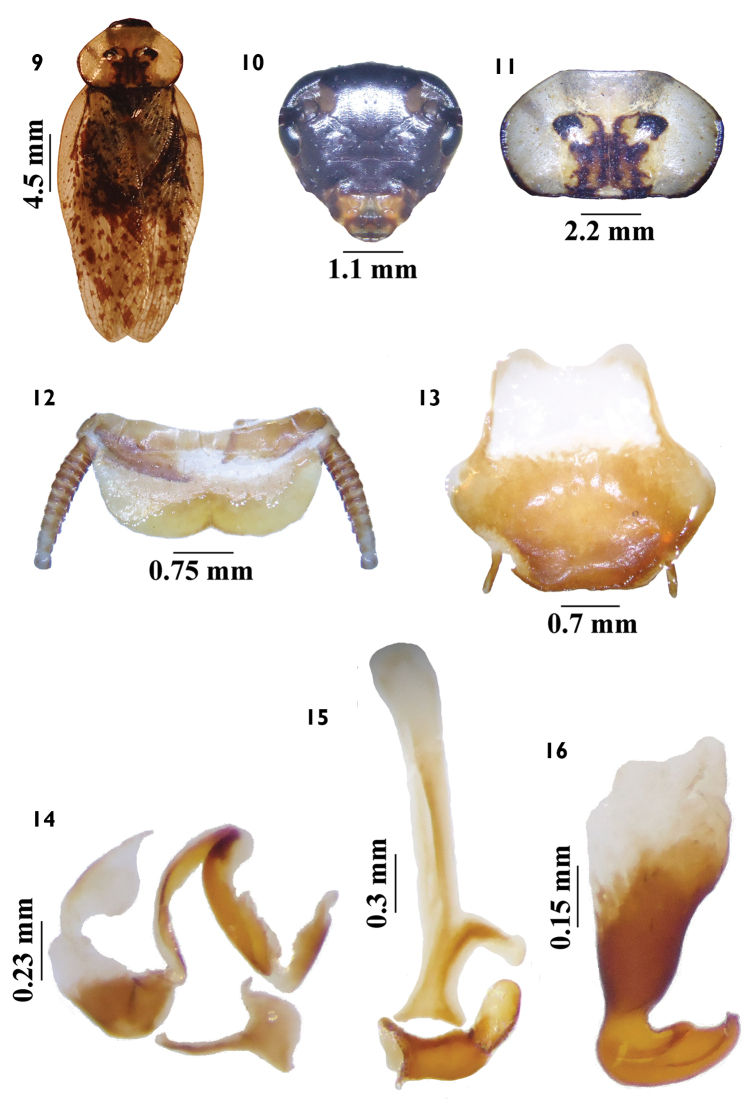
*Tribonium morroferrensis* sp. n. **9** *habitus*, dorsal view **10** head, ventral view **11** pronotum, dorsal view **12** supranal plate, dorsal view **13** subgenital plate, ventral view **14** left phallomere, dorsal view **15** median sclerite, dorsal view **16** right phallomere, dorsal view.

**Male holotype.** Coloration yellowish brown with dark brown spots (Fig. 9). Head black with yellowish brown ocellar and clypeal areas (Fig. 10). Pronotum medially with symmetrical spots, without definite shape (Fig. 11). Tegmina with anal field with brown spots. Marginal and scapular fields light brown with brown spots. Pulvilli light brown. Arolia and claws brown.

**Head.** Triangular, vertex slightly exposed, interocular space ample, 1.9 mm, approximately 2/3 distance between antennae. Antenna not reaching apex of tegmen. Palpi and antennae ciliated; second segment of maxillary palpi small, third segment 0.70 mm in length, fourth segment same size as second, fifth segment clavate, similar in size to third segment, conspicuously ciliated.

**Thorax.** Pronotum convex, transverse, apical surface embossed, with rounded margin and straight basal surface. Tegmina developed, extending beyond apex of cerci; marginal field short and slightly concave; scapular field tapering toward apex, discoidal field extended apically, slightly angular along veins. Wings with marginal field narrow, subcostal vein reaching basal third of scapular field; cubital vein with numerous complete and few incomplete veins; apical triangle absent. Legs short, robust, inner thigh narrow with clear apical projection. Fore, mid and hind femora sparsely ciliated along anteroventral border. Pulvilli small, present on fourth tarsal segments; claws simple, symmetrical, with concave arolia.

**Abdomen.** Supranal plate ciliated, with prominent median edges between enclosures, with slight median invagination; cerci short, ciliated, with 12 segments (Fig. 12). Subgenital plate asymmetrical (Fig. 13). Right phallomere hook-shaped, with subapical cleft (Fig. 14); median sclerite with pre-apical lateral projection (Fig. 15); left phallomere slightly sclerotized (Fig. 16).

#### Diagnosis.

*Tribonium morroferrensis* sp. n. resembles *Tribonium guttulosum* (Walker, 1868) in general coloration and pronotum (Figs 11, 19), but differs in size and coloration of the tegmina (Figs 9, 17), color of head (Figs 10, 18), and genital parts (Figs 14–16, 22–24). The supranal and subgenital plates are blaberoid type (Figs 12–13; 20–21).

**Figures 17–24.**
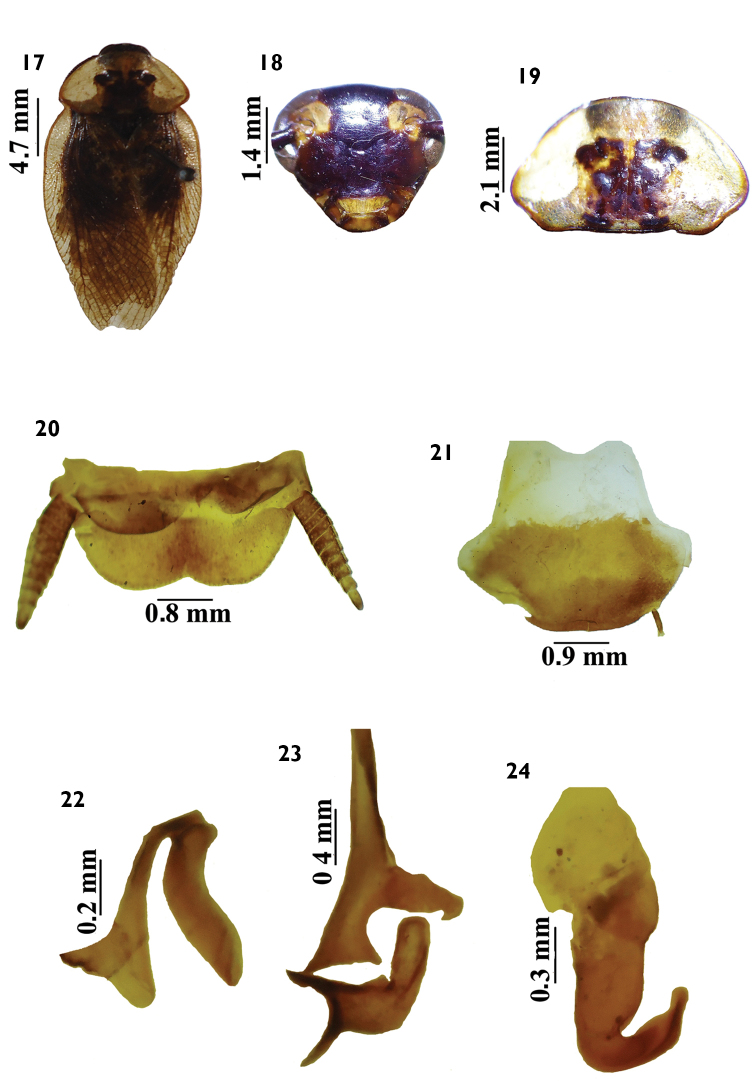
*Tribonium guttulosum* (Walker, 1868). **17** *habitus*, dorsal view **18** head, ventral view **19** pronotum, dorsal view **20** supranal plate, dorsal view **21** subgenital plate, ventral view **22** left phallomere, dorsal view **23** median sclerite, dorsal view **24** right phallomere, dorsal view.

**Figures 25–32.**
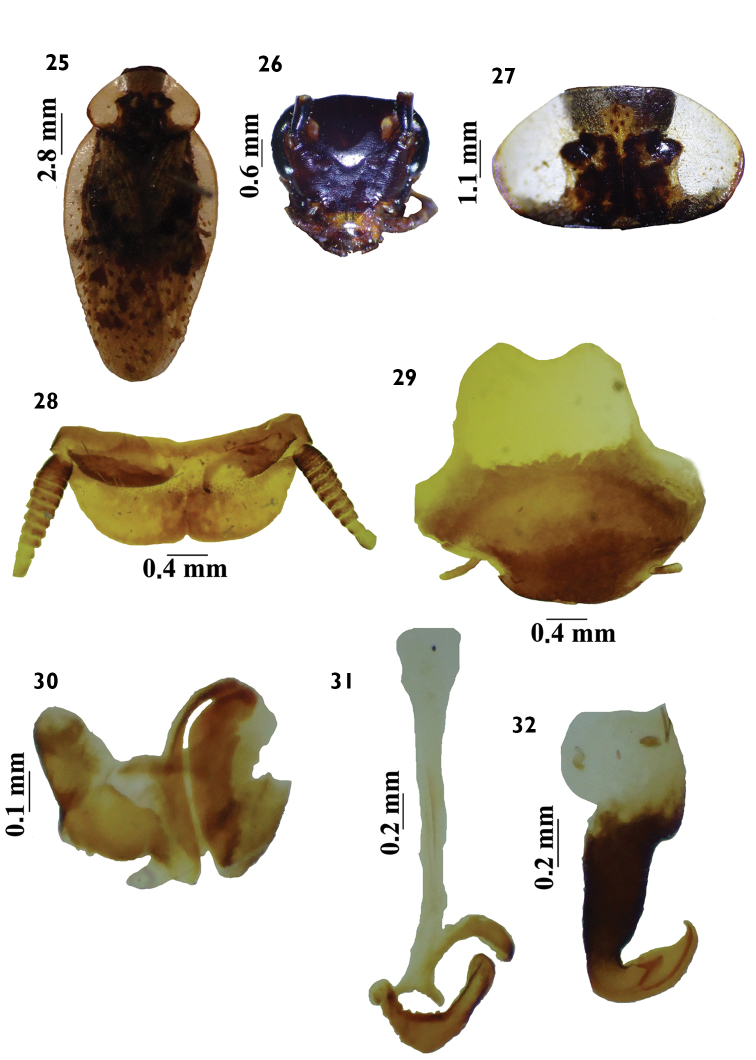
*Tribonium neospectrum* Lopes, 1978. **25** *habitus*, dorsal view **26** head, ventral view **27** pronotum, dorsal view **28** supranal plate, dorsal view **29** subgenital plate, ventral view **30** left phallomere, dorsal view **31** median sclerite, dorsal view **32** right phallomere, dorsal view.

### Key to adult males of the genus *Tribonium*

**Table:** 

1	Total length of specimen 27 mm or more	***Tribonium conspersum* (Guérin-Méneville & Percheron, 1835)**
–	Total length of specimen less than 27 mm	**2**
2	Pronotum with compact, symmetrical dark-brown to black medial spot with no definite shape	**3**
–	Pronotum without compact medial spot	**4**
3	Tegmina without concentration of dark-brown spots at base of discoidal field	***Tribonium delicatum* Lopes, 1978**
–	Tegmina with concentration of dark-brown spots or spots scattered throughout its length	**6**
4	Supranal with tapered paraprocts, cerci long and tapered	***Tribonium litoris* Lopes, 1978**
–	Supranal with well-defined paraprocts, cerci short and wide	**5**
5	Apex of the median sclerite arrow-shaped	***Tribonium sagittum* Lopes & Silva, 2010**
–	Apex of the median sclerite elongated	***Tribonium neospectrum* Lopes, 1978**
6	Head with vertex dark brown with yellowish-brown ocellar and clypeal areas	***Tribonium guttulosum* (Walker, 1868)**
–	Head with vertex black with yellowish-brown ocellar and clypeal areas	**7**
7	Tegmina with the base of discoidal field with spots less concentrated	***Tribonium caldensis* sp. n.**
–	Tegmina with the base of discoidal field with more concentrated spot	***Tribonium morroferrensis* sp. n.**

## Supplementary Material

XML Treatment for Tribonium caldensis

XML Treatment for Tribonium morroferrensis
